# Analysis of the safety and efficacy of single-stage spinal cord stimulation implantation under general anesthesia for high-risk diabetic feet

**DOI:** 10.1016/j.ibneur.2026.07.020

**Published:** 2026-08-06

**Authors:** Xiaochen Chen, Baochang Zhu, Zhitao Li

**Affiliations:** a Department of Neurosurgery, Tianjin Huanhu Hospital, Tianjin Key Laboratory of Cerebral Blood Flow Reconstruction and Head and Neck Tumor New Technology Translation, Tianjin 300350, China; b Department of Diabetic Foot Care, Zhu Xianyi Memorial Hospital of Tianjin Medical University, Tianjin 300400, China; c Department of Neurosurgery, Huanhu Hospital Affiliated to Tianjin Medical University, Tianjin Key Laboratory of Cerebral Blood Flow Reconstruction and Head and Neck Tumor New Technology Translation, Tianjin 300350, China

**Keywords:** Spinal Cord Stimulation, High-risk Diabetic Feet, Critical Limb Ischemia, Pain, Diabetic Peripheral Neuropathy

## Abstract

**Objective:**

To evaluate the safety and efficacy of implantation of a one-stage spinal cord stimulation (SCS) system under general anesthesia in patients with high-risk diabetic feet.

**Methods:**

A retrospective analysis was performed on 25 patients who were diagnosed with high-risk diabetic feet and who underwent implantation of an SCS system between January 2023 and May 2024. The procedure was conducted under general anesthesia with intraoperative electrophysiological monitoring. All patients were followed up for one year postoperatively. The outcomes, including pain intensity, foot skin temperature, dorsalis pedis artery flow velocity, and F-wave occurrence rate on electromyography, were assessed at baseline and at multiple time points after surgery. Additionally, postoperative complications such as infection, electrode displacement, and lead fracture, as well as rates of new ulcer formation, amputation, and mortality, were recorded.

**Results:**

All patients completed the 12-month follow-up period. The efficacy rate was 100% at 7 days postoperatively and 88% at 6 months, and the incidence of new ulcers at 1 year was 8%. Significant improvements in pain, foot skin temperature, and arterial flow velocity were observed within 7 days after implantation (*P* = 0.000), with sustained therapeutic effects lasting beyond 6 months (*P*’=0.000). However, no further improvement in the dorsalis pedis artery flow velocity was noted with prolonged stimulation duration (*P*”=0.459). Electromyography revealed no significant change in the F-wave occurrence rate in the early postoperative phase (*P* = 0.074), but a significant increase was observed at 6 months post-stimulation (*P*’=0.003).

**Conclusion:**

One-stage implantation of an SCS system under general anesthesia is safe and effective for managing high-risk diabetic feet. The system significantly alleviates pain, improves lower extremity perfusion, and enhances nerve conduction function. Notably, while vascular improvements occur rapidly, neurological recovery may require a longer duration of stimulation.

## 1 Introduction

Diabetes is among the most prevalent chronic diseases worldwide (Lazzarini et al., 2018), and diabetic foot is a severe complication characterized by high incidence, amputation, and mortality rates. This condition significantly impairs patients’ quality of life, reduces life expectancy, and increases healthcare costs (Armstrong et al., 2017, Jeffcoate et al., 2018, Peng et al., 2021, Zhang et al., 2020). Current treatment approaches for diabetic foot include vascular recanalization, standard wound care (such as debridement, dressing changes, pressure offloading, and glycemic control), and antimicrobial therapy. However, even with strict adherence to these protocols, fewer than 50% of diabetic foot ulcers achieve healing, and approximately 60% become infected, increasing the risk of amputation by 155-fold following infection (Holman et al., 2015, Pickwell et al., 2015). Research has indicated that the 5-year mortality rate after minor amputation (partial foot) ranges from 29% to 69%, whereas that following major amputation (above the ankle) ranges from 52% to 80% (Pop-Busui et al., 2017, Rastogi et al., 2020, Schmidt et al., 2019). These findings highlight that preventing foot ulcers plays a critical role in reducing both amputation and mortality rates.

Spinal cord stimulation (SCS), a neuromodulation technique, has been increasingly utilized in the management of diabetic foot in recent decades. Clinical evidence has demonstrated that SCS can effectively reduce the amputation rate (Cyrek et al., 2024, Liu et al., 2025, Xu et al., 2023, Yao et al., 2024, Zhou et al., 2024). However, standardized criteria for patient selection are lacking, and long-term clinical outcomes vary according to the severity of the disease. Horsch and Claeys (1994) reported amputation rates of 11% in patients at Fontaine stage III and 70% in those at Fontaine stage IV among individuals with severe lower extremity arterial ischemia who were not candidates for vascular recanalization and received SCS treatment (with a mean follow-up of 35.6 months). Similarly, Petrakis and Sciacca (1999) reported comparable outcomes. These findings indicate that early initiation of SCS in high-risk patients is associated with greater long-term benefits.

Currently, the surgical approach for SCS predominantly involves a two-stage implantation procedure under local anesthesia. In this approach, electrodes are initially implanted for a trial period of 1–2 weeks. If the clinical response is confirmed to be satisfactory, a second surgery is performed to implant the implantable pulse generator (IPG). This staged strategy may lead to a suboptimal patient experience and is associated with increased risks of electrode displacement, fracture, and infection (Zhang et al., 2025). Given that lesion locations in patients are typically stable, the optimal electrode placement segments tend to be consistent across individuals. Therefore, single-stage implantation of both the electrode and the IPG under general anesthesia, with intraoperative electrophysiological monitoring, may reduce the risk of infection and electrode-related complications while improving the overall patient surgical experience.

Patients at high risk of diabetic foot include individuals with diabetes who have risk factors—such as peripheral neuropathy, severe lower extremity ischemia, or foot deformity—but no active foot ulcers and those in whom previous ulcers have fully healed (Bus et al., 2024). Currently, clinical studies evaluating the efficacy of single-stage implantation of an SCS system under general anesthesia with intraoperative electrophysiological monitoring for the management of high-risk diabetic feet are lacking. This study aims to investigate the safety and effectiveness of this surgical approach and to provide evidence-based data to support clinical decision-making regarding patient selection criteria.

## 2 Material and methods

### 2.1 Study design

This study was approved by the Institutional Review Board of Tianjin Huanhu Hospital (Approval Number: 202401311449000500510). All patients provided written informed consent prior to undergoing surgical intervention. Electromyography (EMG), somatosensory-evoked potential (SEEP) monitoring, lower extremity arterial ultrasound, and computed tomography angiography (CTA) were performed both before and after surgery. To ensure the consistency and reliability of the data, all the examinations were conducted by the same physician. Similarly, surgical procedures, postoperative programming, and follow-up assessments were carried out by a single surgeon throughout the study period.

### 2.2 Study Participants

This retrospective study included a total of 25 patients with high-risk diabetic feet who received SCS treatment at Tianjin Huanhu Hospital between January 2023 and May 2024. The inclusion criteria were as follows: (1) age ≥ 18 years, a diagnosis of high-risk diabetic feet (Wagner grade 0; International Working Group on the Diabetic Foot (IWGDF) grade 2–3), and the presence of refractory chronic pain (visual analog scale [VAS] ≥5 cm); (2) severe lower extremity arterial ischemia (Fontaine stage III), unsuitable for or unsuccessful endovascular intervention, with or without peripheral neuropathy; and (3) full understanding of the SCS surgical procedure and provision of written informed consent for treatment and follow-up. The exclusion criteria included (1) the presence of active ulcers or localized gangrene; (2) concomitant malignancy or other severe comorbid conditions associated with an estimated life expectancy of less than two years; and (3) the presence of surgical contraindications or the inability to tolerate general anesthesia. In this study, the primary endpoint was the development of foot ulcers or gangrene, whereas the secondary endpoints included improvements in various clinical parameters.

### 2.3 Surgical procedure

**Fig. 1:**
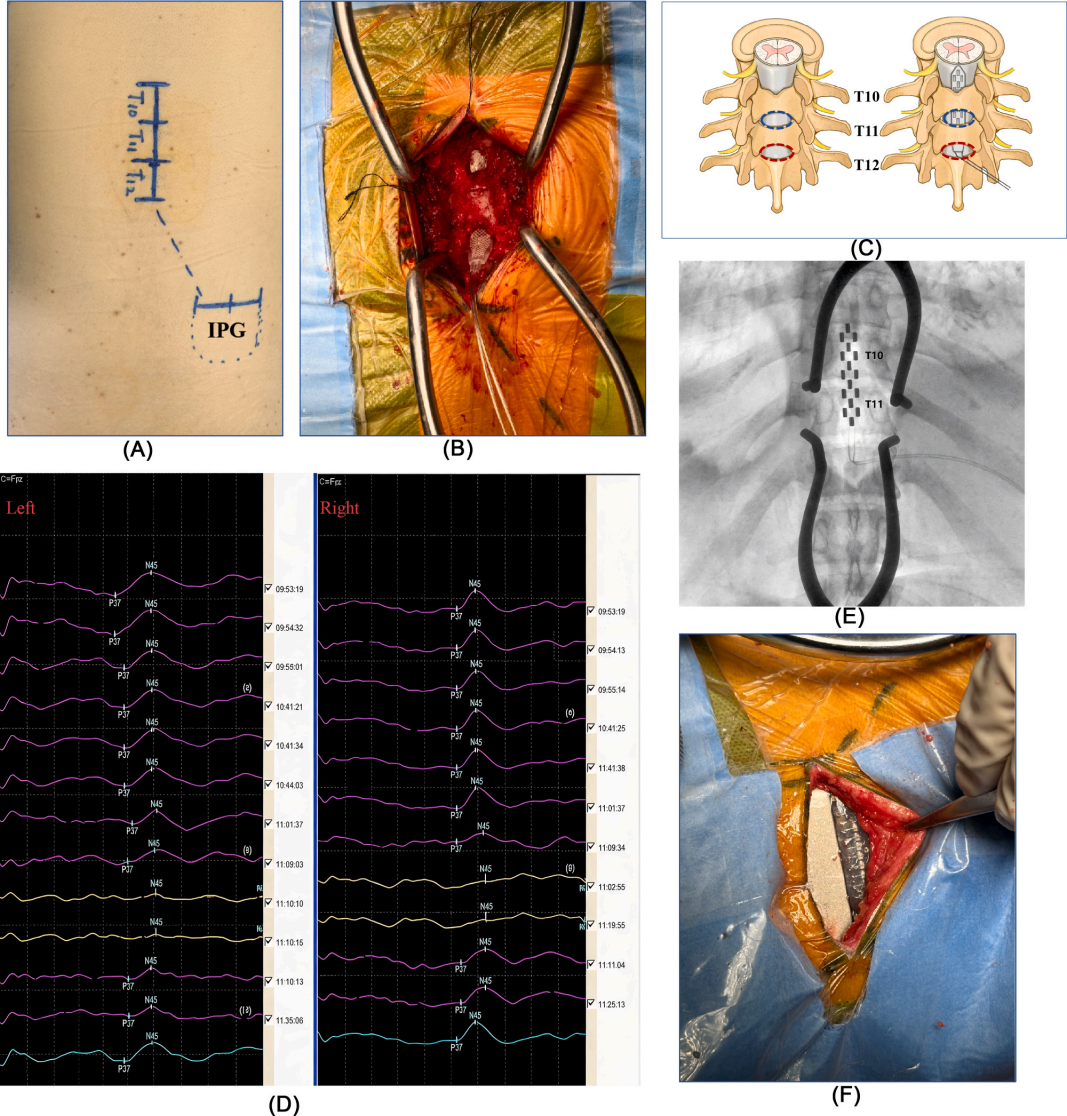
Surgical procedure. (A) Preoperative localization of the T11 vertebra and marking of the planned incision site. The area enclosed by the dashed line and the solid line indicates the placement location of the IPG; (B) Electrode implantation at the T10–11 vertebral level; (C) Schematic illustration of electrode placement: the red oval indicates the T11/12 lamina fenestration, referred to as the “operative window,” through which the stimulation electrode is inserted; the blue oval represents the T10/11 lamina fenestration, termed the “observation window,” used to ensure central alignment of the electrode during implantation; (D) Intraoperative electrophysiological monitoring showed a reversible reduction in the N45 wave amplitude of bilateral somatosensory evoked potentials (SEPs) upon device activation, with recovery observed after deactivation; (E) Following successful intraoperative test stimulation, X-ray imaging was performed to confirm precise electrode positioning; (F) A subcutaneous pocket was created in the right lumbar region for implantation of the implantable pulse generator (IPG).

The surgical procedure involved a single-stage implantation of the SCS system under general anesthesia with intraoperative electrophysiological monitoring. The patients were positioned in the prone position, and fluoroscopic guidance using a C-arm was employed to identify the T11 vertebral level. A posterior midline skin incision was marked and centered on T11. Following the exposure of the spinous processes from T10 to T12, these structures were removed to facilitate access. Laminectomies were performed at the T10/11 and T11/12 interspaces to expose the dura mater. A quadripolar surgical electrode (PINS L3253) was inserted cephalically through the T11/12 interspace, while the T10/11 interspace was utilized for visual assessment of electrode midline positioning. Upon achieving satisfactory paresthesia coverage, the electrode was connected to an external pulse generator for intraoperative test stimulation. Electrophysiological monitoring demonstrated a reversible reduction in the amplitude of N45 somatosensory evoked potentials in both lower limbs during stimulation, with recovery upon cessation, confirming accurate coverage and effective neuromodulation. Fluoroscopic imaging was repeated to verify that the electrode lead was properly positioned within the T10–T11 spinal segment and centrally aligned without lateral deviation. The lead was then secured during implantation of the permanent pulse generator (PINS G122) (Fig. 1).

Following emergence from anesthesia, the device was activated. Stimulation parameters were programmed on the basis of the anatomical distribution of the patient’s pain, including the voltage (0.7–2.55 V), frequency (20–60 Hz), and pulse width (180–300 µs), to achieve complete paresthesia coverage of the affected lower limb without inducing discomfort. During subsequent follow-up visits, a designated physician adjusted the stimulation settings according to the individual clinical response and symptom progression.

### 2.4 Clinical assessment

Clinical assessment was conducted using the visual analog scale (VAS) to evaluate pain intensity. Infrared thermal imaging was employed to assess the skin temperature of the affected foot. Six standardized anatomical sites (the hallux, fifth toe, medial dorsum of the foot, lateral dorsum of the foot, medial malleolus, and lateral malleolus) were selected for measurement, and the mean skin temperature was computed. Ultrasound, which is one of the most widely utilized noninvasive modalities for vascular assessment, enables the measurement of the arterial diameter, degree of stenosis, and flow velocity and provides insight into regional hemodynamic status. In this study, lower extremity arterial ultrasound was performed to evaluate foot perfusion by measuring the flow velocity in the dorsalis pedis artery. F-waves are low-amplitude antidromic action potentials elicited following supramaximal stimulation of the nerve trunk, appearing after the muscle action potential and reflecting conduction integrity along the proximal segment of the peripheral nerve. The normal F-wave persistence rate ranged from 80% to 100%, with a reduced rate indicating proximal nerve dysfunction or demyelinating lesions. Data were collected at three time points—preoperatively, 7 days postoperatively, and 6 months postoperatively—by a designated physician, and changes over time were analyzed. Treatment efficacy was defined as reductions of more than 50% in pain scores and ulcer formation following surgery.

### 2.5 Follow-up

All patients were followed up for one year. The follow-up information included whether new ulcers were present, pain recurred, infection occurred in the surgical area, electrode displacement occurred, lead breakage occurred, and complications such as amputation and death occurred.

### 2.6 Statistical methods

Statistical analysis and image processing were conducted using GraphPad Prism 9.5（GraphPad Software,USA）. Continuous variables are presented as the mean±standard deviation, while categorical and ordinal variables are expressed as percentages. Data normality was assessed using Q–Q plots. For continuous variables following a normal distribution, one-way ANOVA was used to compare the dorsalis pedis artery flow velocity, VAS score and skin temperature at three time points: preoperatively, 7 days postoperatively, and 6 months postoperatively. For data with a nonnormal distribution, the Friedman test was applied. P < 0.05 was considered to indicate statistical significance.

## 3 Results

### 3.1 Demographic characteristics

A total of 25 patients were included in this study, including 18 males (72%) and 7 females (28%), with a mean age of 69.24 ± 8.23 years. Peripheral neuropathy was observed in 17 patients (68%), and 9 patients (36%) had a history of lower extremity ulcers, all of which had healed prior to surgery. Additional demographic and clinical characteristics are summarized in Table 1. All patients completed the one-year follow-up period. No major complications, including infection, electrode displacement, lead fracture, or mortality, were reported during the follow-up period. The treatment effectiveness rate was 100% at 7 days postoperatively, decreased to 88% at 6 months, and remained stable thereafter. A new ulceration rate of 8% was observed at one year, with all new ulcers occurring within 6 months after the procedure. The detailed outcomes for treatment effectiveness are presented in Table 2.

**Table 1:** Patient demographic characteristics.

Age, y	69.24 ± 8.28
Course of disease, m	36.60 ± 58.77
Sex, n(%)	
Male	18(72.0)
Female	7(28.0)
Affected side, n(%)	
Left	6(24.0)
Right	3(12.0)
Bilateral	16(64.0)
Hypertension, n(%)	14(56.0)
History of smoking, n(%)	13(52.0)
History of lower extremity ulcers	9(36.0)
Peripheral neuropathy	17(68.0)

**Table 2:** Therapeutic effects of SCS at different time points.

	Pre-op	7 days post-op	*P*	6 months post-op	*P’*	1 year post-op
VAS	7.04 ± 0.93	1.72 ± 1.02	0.000	1.68 ± 1.25	0.000	
Skin temperature, °C	28.81 ± 1.27	30.04 ± 0.87	0.000	30.17 ± 0.90	0.000	
Dorsalis pedis artery flow velocity （cm/s）	20.63 ± 17.48	30.46 ± 23.45	0.000	32.50 ± 21.92	0.000	
Occurrence rate of F-waves (%)	25.00 ± 28.76	63.75 ± 40.52	0.074	68.33 ± 42.23	0.003	
Complication						
Infection, n	0	0		0		0
Electrode displacement, n	0	0		0		0
Ulcers, n（%）	0	0		2（8.0）		2
Amputation, n	0	0		0		0
Death	0	0		0		0
Effective rate （%）		100		88.0		88

### 3.2 Pain Scores

**Fig. 2:**
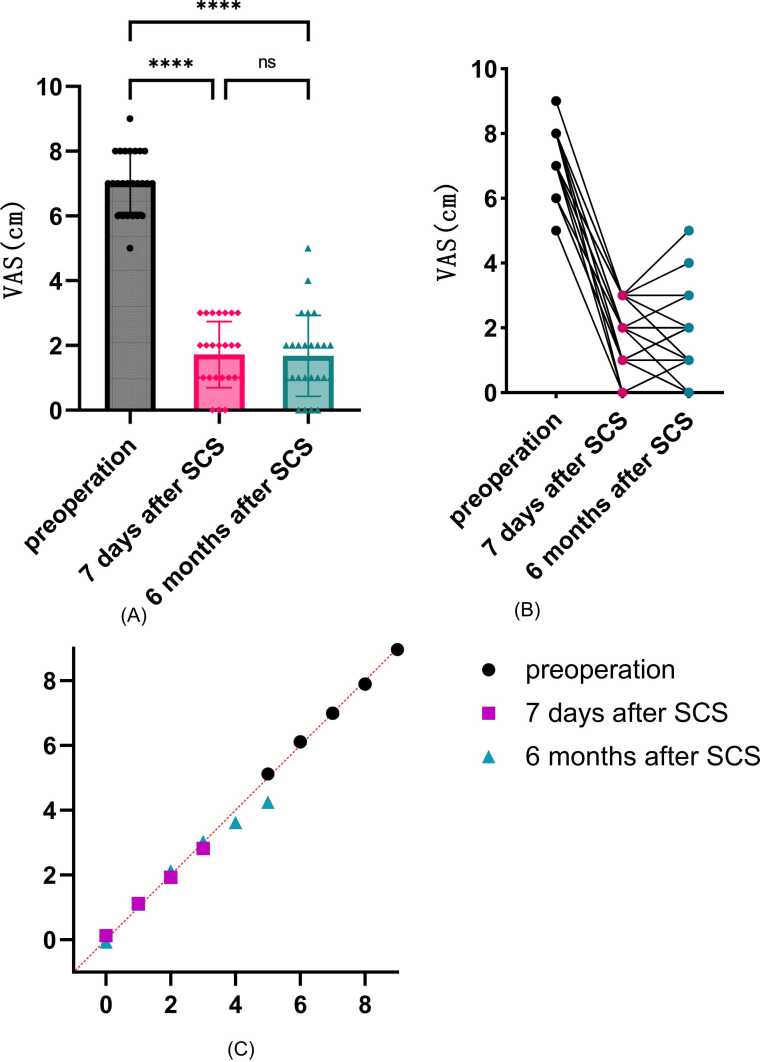
Visual analog scale (VAS) scores at various time points before and after surgery. (A) Comparison of VAS scores prior to spinal cord stimulation (SCS) treatment with those at 7 days and 6 months post-treatment; (B) Individual patient trajectories of VAS changes across different time points; (C) Q—Q plot confirming nonnormal distribution of the data. ****P < 0.0001.

The VAS is a widely used instrument for assessing pain intensity, ranging from 0 to 10 cm, where 0 cm denotes no pain and 10 cm indicates the worst imaginable pain. The preoperative mean VAS score was 7.04 ± 0.93 cm (n = 25), which decreased to 1.72 ± 1.02 cm at 7 days postoperatively and further stabilized at 1.68 ± 1.25 cm at 6 months postoperatively. Normality assessment was conducted using the Shapiro–Wilk test and Q–Q plots, confirming that all three datasets deviated from a normal distribution. Given the nonparametric nature of the data, the Friedman test was employed to evaluate differences across the three time points. The results revealed a significant reduction in pain scores at both 7 days and 6 months postoperatively compared with those at baseline (*P=*0.000; *P*’=0.000). No significant difference was observed between pain scores at 7 days and 6 months postoperatively (*P*’’=1.000) (Fig. 2).

### 3.3 Skin temperature

**Fig. 3:**
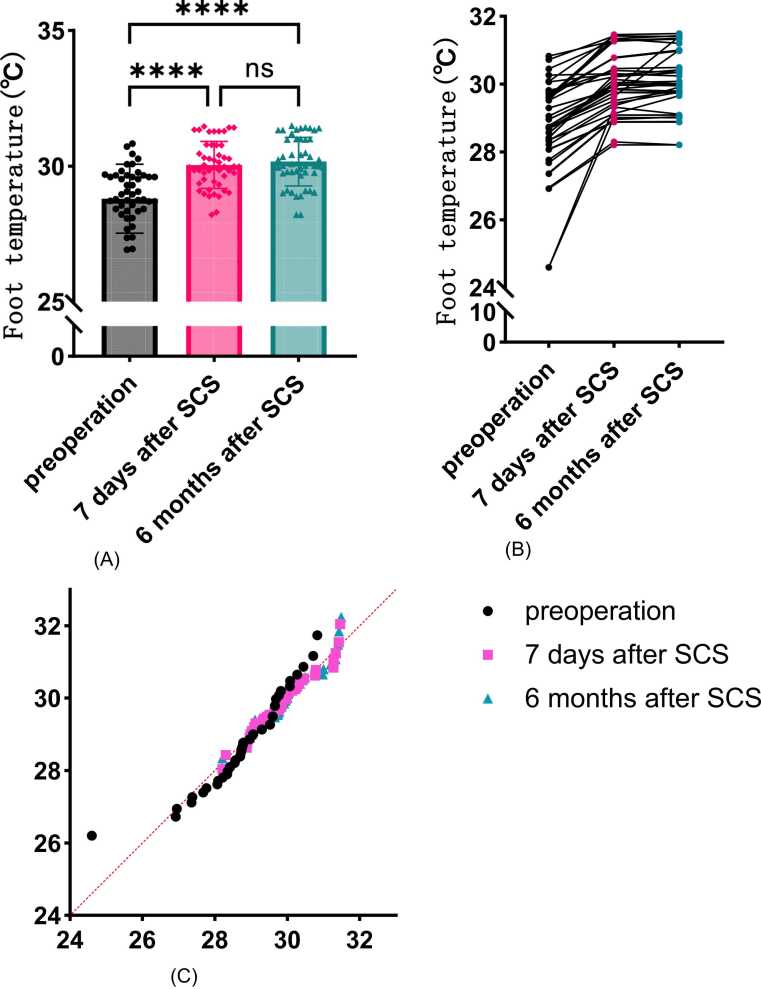
Plantar skin temperature of the affected foot at various time points before and after surgery. (A) Comparison of plantar skin temperature prior to spinal cord stimulation (SCS) treatment with values measured at 7 days and 6 months post-treatment; (B) Q—Q plot confirming the nonnormal distribution of the data. ****P < 0.0001.

The average preoperative skin temperature was 28.81 ± 1.27°C (n = 48), which increased to 30.04 ± 0.87°C on postoperative day 7 and reached 30.17 ± 0.90°C at the 6-month follow-up. The normality of the data was evaluated using the Kolmogorov–Smirnov test and Q–Q plots, indicating that skin temperature measurements before surgery and at 6 months postoperatively conformed to a normal distribution, whereas those on postoperative day 7 deviated from a normal distribution. To examine differences across the three time points, the Fridman test was conducted. The results revealed statistically significant increases in skin temperature on postoperative day 7 and at 6 months compared with the preoperative values (*P* = 0.000, *P*’=0.000), with no significant difference between day 7 and 6 months postoperative (*P*”=0.553) (Fig. 3).

### 3.4 Dorsalis pedis artery flow velocity

**Fig. 4:**
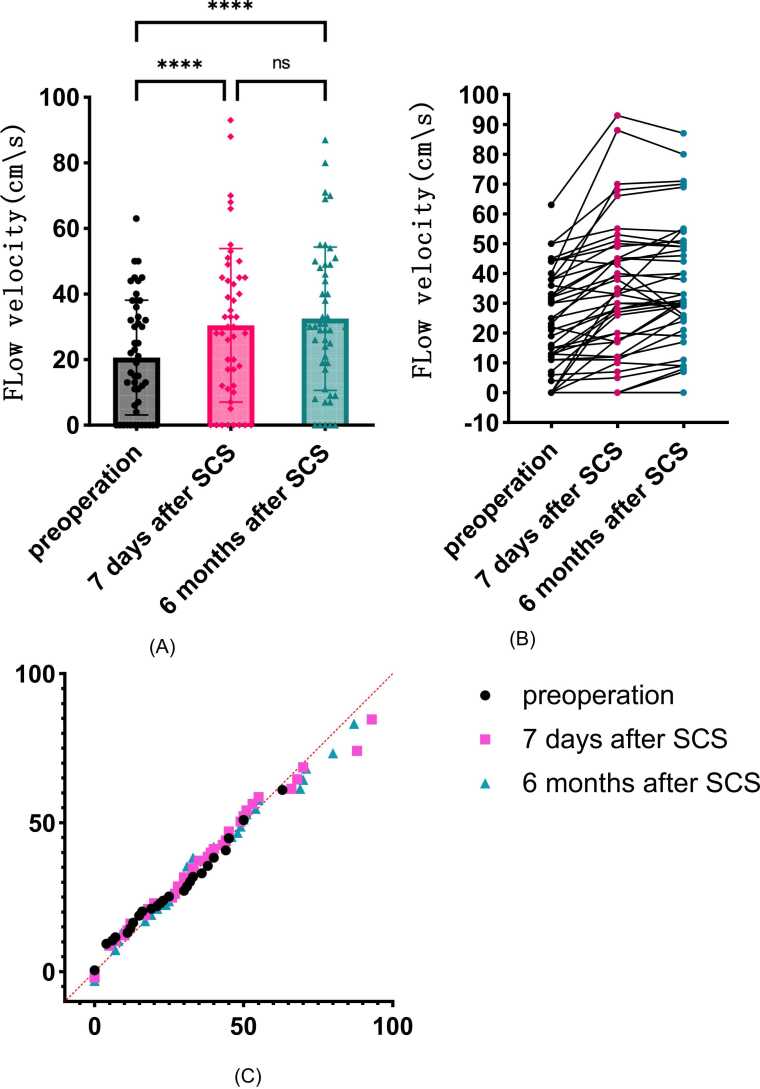
Flow velocity of the dorsalis pedis artery at various time points before and after surgery. (A) Comparison of the dorsalis pedis artery flow velocity prior to spinal cord stimulation (SCS) treatment with measurements obtained at 7 days and 6 months post-treatment; (B) Individual patient trajectories of flow velocity changes over time; (C) Q—Q plot confirming the nonnormal distribution of the data. ****P < 0.0001.

The mean preoperative flow velocity was 20.63 ± 17.48 cm/s (n = 48), which increased to 30.46 ± 23.45 cm/s on postoperative day 7 and reached 32.50 ± 21.92 cm/s at 6 months, following a normal distribution, whereas the preoperative values were not normally distributed. The Friedman test was applied to compare flow velocity across the three time points. The results demonstrated that the flow velocity in the dorsalis pedis artery was significantly greater on day 7 and at 6 months postoperatively than that preoperatively (*P=*0.000, *P*’=0.000), with no statistically significant difference between the two postoperative time points (*P*”=0.459) (Fig. 4).

### 3.5 F-waves

**Fig. 5:**
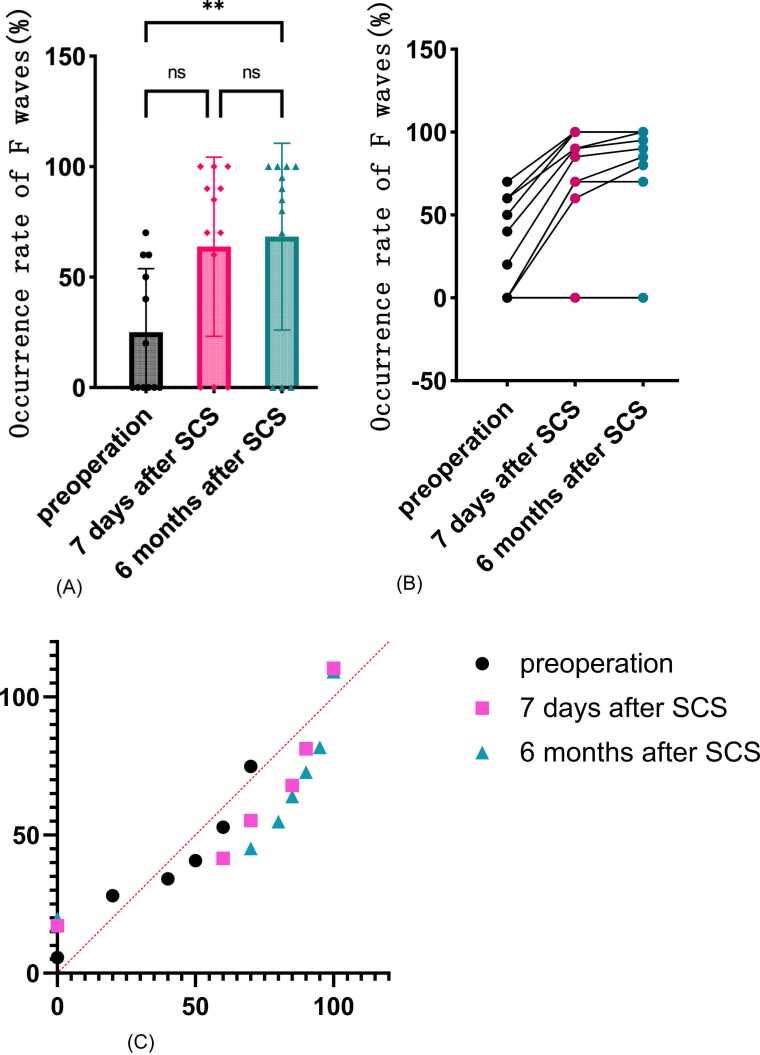
F-wave persistence rate at various time points before and after surgery. (A) Comparison of the F-wave persistence rate prior to spinal cord stimulation (SCS) treatment with values measured at 7 days and 6 months post-treatment; (B) Q—Q plot confirming the nonnormal distribution of the data. **P < 0.01.

In this study, 17 patients were diagnosed with concomitant peripheral nerve involvement, 12 of whom completed electromyographic follow-up assessments. The mean F-wave persistence rate was 25.00%±28.76% preoperatively, increased to 63.75%±40.52% on postoperative day 7, and reached 68.33%±42.23% at the 6-month follow-up. Normality was assessed using the Shapiro–Wilk test and Q–Q plots, which confirmed that all three time points deviated from a normal distribution. The Friedman test was employed to evaluate changes across the three measurement periods. No statistically significant difference in F-wave persistence was observed between preoperative and postoperative day 7 values (*P* = 0.074). However, the persistence rate at 6 months postoperatively was significantly greater than that at baseline (*P*’=0.003) (Fig. 5).

## 4 Discussion

SCS for chronic pain management typically involves a two-stage surgical approach: an trial stimulation phase using percutaneous leads to assess therapeutic efficacy prior to permanent implantation of IPG,which will increase the risk of electrode-related infections due to long-term exposure. Moreover, cylindrical percutaneous electrodes—commonly employed during trial stimulation—are susceptible to intraoperative and postoperative migration, potentially resulting in false-negative outcomes. In contrast, single-stage implantation of paddle-type electrodes under general anesthesia enables broader and more stable dorsal column coverage, with reduced risk of lead displacement. When integrated with intraoperative electrophysiological monitoring—including somatosensory evoked potentials (SSEPs) and motor-evoked potentials (MEPs)—this approach facilitates precise anatomical targeting of the nociceptive pathways responsible for the patient’s pain distribution. Nevertheless, this single-stage strategy entails several clinical and logistical considerations, including stringent patient selection criteria (e.g., adequate cardiopulmonary reserve), prolonged operative duration, and substantially higher procedural and resource-related costs. This study retrospectively evaluated the clinical efficacy of single-stage implantation of an SCS system under general anesthesia for the treatment of high-risk diabetic feet using intraoperative electrophysiological monitoring. The findings demonstrated that the general anesthesia protocol was safe and reliable, with no major complications—such as infection, electrode displacement, lead fracture, or mortality—observed during the one-year follow-up period. The overall complication rate was lower than that of previously reported outcomes of staged procedures performed under local anesthesia (Liu et al., 2024, Piedade et al., 2023, Ubbink and Vermeulen, 2013). One year after surgery, the new ulceration rate was 8%, and the amputation-free survival rate reached 100%, which is consistent with the results of prior studies (Piedade et al., 2023). The results demonstrated that during the early postoperative period, patients exhibited statistically significant improvements in VAS scores, skin temperature, and posterior tibial artery blood flow velocity compared with preoperative baseline values. Notably, these improvements plateaued beyond a certain stimulation duration. In contrast, the restoration of neural conduction function necessitates a prolonged stimulation duration.

Lower extremity pain in patients with diabetes is often complex and multifactorial and potentially involves both neuropathic and ischemic components simultaneously. SCS has been established as an effective therapeutic option for chronic intractable pain, including neuropathic pain and severe ischemic pain in the lower extremities (Deer et al., 2021). The precise mechanism of action remains incompletely understood; however, several mechanisms have been proposed (Zhang et al., 2025): (1) the gate control theory, where activation of large-diameter Aβ fibers inhibits nociceptive transmission through C fibers; (2) enhancement of inhibitory neurotransmitter release (e.g., GABA, serotonin, and endogenous opioids), suppression of excitatory neurotransmitter release (e.g., glutamate and aspartate), and subsequent blockade of pain signal propagation; (3) reduction in proinflammatory cytokines (such as IL-1β, IL-6, and TNF-α), leading to inhibition of nociceptive Aδ and C fibers; and (4) stimulation of vasodilatory substance release, decreased sympathetic nervous system activity, and reduced catecholamine secretion, thereby ameliorating peripheral ischemia. Previous studies have reported that the efficacy rate of SCS in managing diabetes-related lower extremity pain at 6 months ranges from 73% to 95% (Petersen et al., 2022, Petersen et al., 2021, Petrakis and Sciacca, 1999, Petrakis and Sciacca, 2000, Schnapp and Delcroix, 2022). In this study, the response rate was 88% at one year, which is generally consistent with previous reports. Three patients experienced pain recurrence within six months after surgery, and symptom improvement remained limited despite the optimization of stimulation parameters. All three had a history of prior failed revascularization procedures, and two developed recurrent ulcers. It is hypothesized that the persistent pain in these patients may have resulted from severe underlying arterial occlusion.

**Fig. 6:**
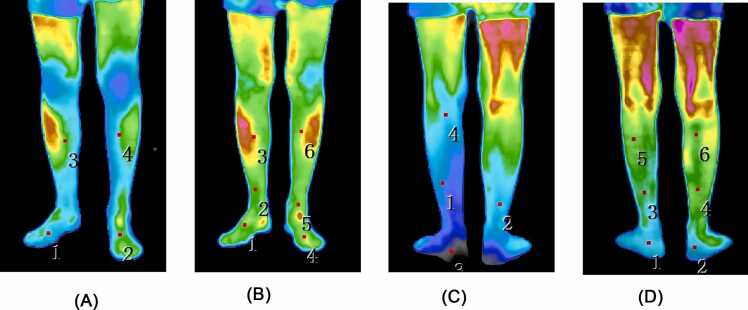
Infrared thermal imaging demonstrating increased skin temperature in both lower extremities following surgery. (A, C) Preoperative images; (B, D) Postoperative images at 7 days after intervention show that the skin temperatures of the bilateral lower limbs significantly increased compared with those before surgery.

**Fig. 7:**
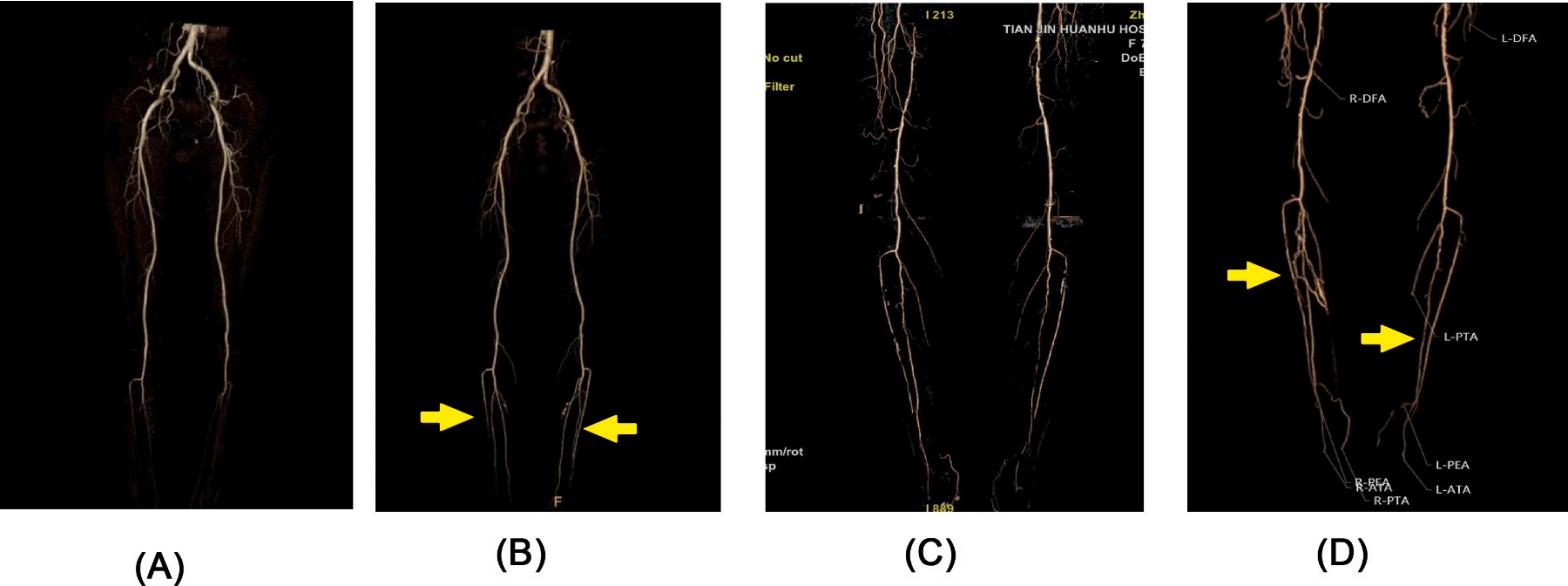
Follow-up lower extremity arterial computed tomography angiography (CTA) performed 6 months after SCS demonstrated a marked improvement in the visualization of the bilateral infrapopliteal arteries(yellow arrowhead) compared with the preoperative images. (A, C) Preoperative images; (B, D) Postoperative images at 6 months after intervention.

Compared with nondiabetic patients with severe lower extremity arterial ischemia, diabetic patients are more likely to exhibit arterial involvement below the knee (Mills, 2016) and typically present with a broader lesion extent (Chung, 2017). Consequently, vascular recanalization is technically more challenging and is associated with longer procedural durations, higher rates of postoperative reocclusion, and an increased risk of arterial dissection and rupture because of repeated endovascular interventions (Tummala et al., 2020). The International Society for Vascular Surgery and other professional organizations published the “Global vascular guidelines on the management of chronic limb-threatening ischemia”, which recommend SCS as a therapeutic option for patients with pain at rest or minor tissue loss who are not candidates for revascularization (Conte et al., 2019). A retrospective observational study demonstrated that the SCS group outperformed the endovascular treatment group in terms of improving skin temperature and enhancing lower extremity arterial dilation (Xu et al., 2023). In this study, the results revealed that SCS significantly increased the lower extremity skin temperature (Fig. 6) and improved the dorsalis pedis artery flow velocity shortly after surgery. Compared with baseline, follow-up lower extremity CT angiography at six months revealed markedly improved patency and visualization of the infrapopliteal arteries (Fig. 7), which likely contributed to the enhanced distal flow. However, no further improvement was observed with prolonged stimulation duration. This plateau effect may be attributable to inherent characteristics of diabetic peripheral vascular disease, such as altered arterial wall mechanics, increased stiffness, and reduced elasticity (Tsuchiya et al., 2005).

Diabetic peripheral neuropathy (DPN) is the most significant risk factor associated with diabetic foot ulcers, with approximately 90% of such ulcers occurring in patients with concomitant DPN (Bowling et al., 2015). Research has indicated that SCS can improve peripheral nerve conduction (Cui et al.,2025). Evidence indicates that although the SCS does not alter the density of peripheral epidermal nerve fibers, it enhances the processing of sensory information in the spinal cord by modulating the excitability of dorsal horn neurons, thereby normalizing certain sensory responses (Wang et al., 2024), which may explain the improvement in the F-wave appearance rate.

This study has several limitations. First, it is a retrospective observational study rather than a prospective randomized controlled trial, which may introduce selection bias and limit the strength of causal inference. Second, the sample size was relatively small, and the follow-up duration was short, potentially affecting the statistical power and generalizability of the findings. In future research, we plan to conduct a multicenter randomized controlled trial with an expanded sample size, a longer follow-up period, and a more comprehensive assessment of microcirculatory parameters, such as the transcutaneous partial pressure of oxygen, as well as a detailed evaluation of improvements in individual lower extremity arteries. These enhancements will allow for a more precise characterization of disease progression and clinical outcomes in patients with high-risk diabetic feet following SCS treatment.

## 5 Conclusions

Single-stage implantation of an SCS system under general anesthesia with intraoperative electrophysiological monitoring is safe and reliable. This intervention can significantly increase foot skin temperature and enhance blood perfusion while effectively alleviating pain. It is particularly suitable for patients with high-risk diabetic foot at Fontaine stage III who are not candidates for vascular recanalization procedures. The SCS system can improve lower extremity blood flow in the short term, with benefits sustained for more than 6 months, thereby delaying disease progression and improving quality of life. Notably, if there is no substantial improvement in blood flow shortly after SCS initiation, the likelihood of meaningful recovery in the later stages is low, and prolonged stimulation does not lead to further hemodynamic enhancement.

## Ethics Approval

This study strictly adheres to ethical guidelines. All the participants provided informed consent voluntarily. Personal data were kept confidential and used only for research purposes. The study protocol was approved by the institutional ethics committee. Any potential risks to participants were minimized.

## Compliance with Ethical Standards

None

## Ethical Approval

This study was performed in line with the principles of the Declaration of Helsinki. Approval was granted by the Institutional Review Board of Tianjin Huanhu Hospital (Approval No. 202401311449000500510).

## Informed Consent

Written informed consent was obtained from all individual participants included in the study prior to enrollment and surgical intervention.

## Clinical Trial Registration

Not applicable (this is a retrospective observational clinical study).

## Human and Animal Rights

This study involves only human participants and does not include any animal experiments. All procedures were conducted in accordance with relevant ethical guidelines and regulations.

## Informed consent

Informed consent was obtained from all individual participants included in the study. All participants were fully informed about the purpose, procedures, potential risks, benefits, and the right to withdraw from the study at any time without penalty before signing the written informed consent form. The study protocol was approved by the Ethics Committee of Tianjin Huanhu Hospital (Approval No. 202401311449000500510), and all procedures were conducted in accordance with the Declaration of Helsinki.

## Author contributions

Xiaochen Chen was responsible for patient data collection and analysis, manuscript drafting, and patient management. Baochang Zhu participated in patient screening and evaluation and contributed to the data acquisition. Zhitao Li conceived the study design, performed the surgical procedures, and critically revised the manuscript. All the authors approved the final version of the manuscript.

## Funding

This work was supported by the Tianjin Key Medical Discipline Construction Project (Grant No. TJYXZDXK−3–002A). The funder had no role in the design of the study, data collection, analysis, interpretation of data, or writing of the manuscript.

## CRediT authorship contribution statement

**Baochang Zhu:** Supervision, Project administration, Methodology, Data curation. **Zhitao Li:** Writing – review & editing, Visualization, Supervision, Project administration, Methodology, Investigation. **Xiaochen Chen:** Writing – original draft, Formal analysis, Data curation.

## Conflict of Interest

The authors declare that they have no known competing financial interests or personal relationships that could have appeared to influence the work reported in this paper. All authors certify that no funding or commercial support was received that might constitute a conflict of interest related to this study.
